# Chlorido(1,2-dimethyl-1*H*-imidazole-κ*N* ^3^){2-[(diphen­oxy­phosphan­yl)­oxy]phenyl-κ^2^ *C* ^1^,*P*}palladium(II)

**DOI:** 10.1107/S1600536812003431

**Published:** 2012-01-31

**Authors:** Izabela Błaszczyk, Anna M. Trzeciak, Andrzej Gniewek

**Affiliations:** a Faculty of Chemistry, University of Wrocław, 14 F. Joliot-Curie, 50-383 Wrocław, Poland

## Abstract

The Pd atom in the title compound, [Pd(C_18_H_14_O_3_P)Cl(C_5_H_8_N_2_)], adopts a slightly distorted square-planar coordination geometry, with the metallated carbon positioned *trans* to the Cl atom. The crystal structure is stabilized by several weak C—H⋯O and C—H⋯Cl hydrogen-bond inter­actions. One of the phenyl rings is disordered over two almost equally occupied sites.

## Related literature

The structure of the title compound was determined as part of a larger study on orthopalladated triaryl­phosphite complexes. For related structures and further discussion, see: Albinati *et al.* (1990 ▶); Błaszczyk *et al.* (2011 ▶). For the catalytic reactions, see: Miyaura *et al.* (1981 ▶); Sonogashira *et al.* (1975 ▶). For bond lengths in coordination complexes, see: Orpen *et al.* (1989 ▶). For hydrogen-bond inter­actions, see: Aullón *et al.* (1998 ▶); Desiraju & Steiner (1999 ▶). For details of the temperature control applied during data collection, see: Cosier & Glazer (1986 ▶); and for specifications of analytical numeric absorption correction, see: Clark & Reid (1995 ▶).
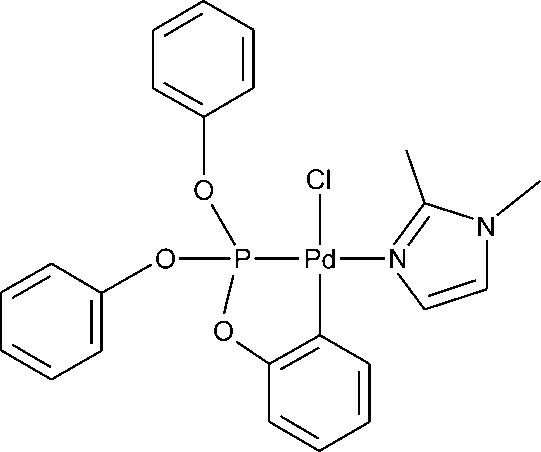


## Experimental

### 

#### Crystal data

[Pd(C_18_H_14_O_3_P)Cl(C_5_H_8_N_2_)]
*M* *_r_* = 547.25
Triclinic, 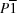

*a* = 9.212 (4) Å
*b* = 9.370 (4) Å
*c* = 14.295 (5) Å
α = 85.30 (3)°
β = 84.83 (3)°
γ = 69.08 (3)°
*V* = 1146.2 (8) Å^3^
*Z* = 2
Mo *K*α radiation
μ = 1.02 mm^−1^
*T* = 100 K
0.34 × 0.16 × 0.12 mm

#### Data collection

Kuma KM-4 CCD diffractometer
Absorption correction: analytical (*CrysAlis RED*; Oxford Diffraction, 2010 ▶) *T*_min_ = 0.812, *T*_max_ = 0.908
12801 measured reflections
5113 independent reflections
4855 reflections with *I* > 2σ(*I*)
*R*_int_ = 0.023

#### Refinement

*R*[*F* ^2^ > 2σ(*F* ^2^)] = 0.029
*wR*(*F* ^2^) = 0.082
*S* = 1.37
5113 reflections
319 parameters
H-atom parameters constrained
Δρ_max_ = 0.39 e Å^−3^
Δρ_min_ = −0.40 e Å^−3^

### 

Data collection: *CrysAlis CCD* (Oxford Diffraction, 2010 ▶); cell refinement: *CrysAlis RED* (Oxford Diffraction, 2010 ▶); data reduction: *CrysAlis RED*; program(s) used to solve structure: *SHELXS97* (Sheldrick, 2008 ▶); program(s) used to refine structure: *SHELXL97* (Sheldrick, 2008 ▶); molecular graphics: *ORTEP-3* (Farrugia, 1997 ▶); software used to prepare material for publication: *SHELXL97*.

## Supplementary Material

Crystal structure: contains datablock(s) global, I. DOI: 10.1107/S1600536812003431/bt5799sup1.cif

Structure factors: contains datablock(s) I. DOI: 10.1107/S1600536812003431/bt5799Isup2.hkl

Additional supplementary materials:  crystallographic information; 3D view; checkCIF report

## Figures and Tables

**Table:** 

Pd—C11	2.003 (3)
Pd—N41	2.088 (3)
Pd—P	2.1667 (12)
Pd—Cl	2.3890 (12)

**Table:** 

P—Pd—Cl	94.52 (4)
P—Pd—C11	81.51 (10)
C11—Pd—N41	93.91 (12)
N41—Pd—Cl	89.88 (8)
C11—Pd—Cl	175.48 (8)
N41—Pd—P	173.55 (8)

**Table 2:** Hydrogen-bond geometry (Å, °)

*D*—H⋯*A*	*D*—H	H⋯*A*	*D*⋯*A*	*D*—H⋯*A*
C14—H14⋯O3^i^	0.95	2.66	3.365 (4)	132
C15—H15⋯Cl^i^	0.95	2.80	3.720 (4)	162
C33—H33⋯Cl^ii^	0.95	2.88	3.541 (4)	128
C35—H35⋯Cl^iii^	0.95	2.89	3.817 (4)	164
C44—H44⋯Cl^iv^	0.95	2.78	3.651 (4)	154

Symmetry codes: (i) 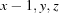; (ii) 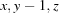; (iii) 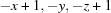; (iv) 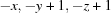.

**Fig. 1.**
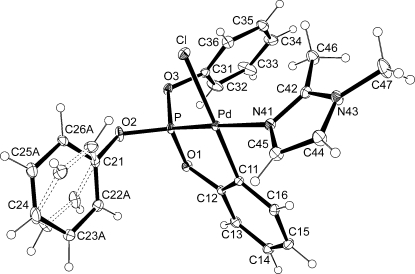
The molecular structure and atom numbering scheme of the title compound. Displacement ellipsoids are drawn at the 30% probability level and H atoms are shown as small spheres of arbitrary radii. Disordered parts with lower occupancy are represented by dashed lines.

## supplementary crystallographic information

### Comment

Carbon-carbon bond-forming catalytic reactions are very important fundamental processes in synthetic chemistry. Among them, the commonly recognized are the Suzuki-Miyaura reaction that leads to formation of biphenyl derivates (Miyaura *et al.*, 1981) and the Sonogashira reaction, which produces phenylated alkines (Sonogashira *et al.*, 1975). In this paper we report the crystallization of a palladacyclic triphenylphosphite complex with a 1,2-dimethylimidazole ligand, the title compound. This complex exhibited high catalytic activity in the Suzuki-Miyaura reaction in ethylene glycol and in the Sonogashira reaction in ionic liquids (Błaszczyk *et al.*, 2011).

The molecular structure of the title compound reveals that the coordination environment of the Pd atom is a slightly distorted square-planar (Fig. 1). The angles between adjacent ligands deviate somewhat from the expected value of 90° (Table 1). However, the observed bond distances and angles are similar to the already reported for analogous complexes (Błaszczyk *et al.*, 2011). In the title compound the metallated carbon is in the *trans* orientation to the Cl atom. As a result of the *trans* influence of the aryl group, the Pd—Cl bond length (Table 1) appears somewhat longer than expected for palladium complexes: 2.298–2.354Å (Orpen *et al.*, 1989). The imidazole ring is oriented at 75.7° with respect to the coordination plane of palladium. The C21—C26 phenyl ring is disordered over two positions with site occupancy factors about 51 and 49%.

The structure is stabilized by a few weak hydrogen bonds of the C—H···O and C—H···Cl types (Desiraju & Steiner, 1999). Consequently, a three-dimensional network of such interactions is formed in the crystal. The shortest C—H···Cl distances observed in the title compound are similar to the values of the N—H···Cl hydrogen bonds identified for Cl bonded to a transition metal (Aullón *et al.*, 1998).

### Experimental

The title compound was prepared according to the previously reported procedure (Błaszczyk *et al.*, 2011): 1,2-dimethylimidazole (0.128 g, 1,32 mmol) was added to the solution of [PdCl{P(OC_6_H_4_)(OC_6_H_5_)_2_}]_2_ (0.30 g, 0.33 mmol) in dichloromethane (4 ml). The starting dimeric palladyclic complex [PdCl{P(OC_6_H_4_)(OC_6_H_5_)_2_}]_2_ had been obtained according to published method (Albinati *et al.*, 1990). The solution was stirred at room temperature for 1 h. The solvent was evaporated *in vacuo* and the white product was precipitated by addition of ethanol and recrystallized from a mixure of dichloromethane and ethanol. Yield: 0.35 g, 97%. Analysis calculated: C 50.48, H 4.05, N 5.12%; found: C 50.30, H 3.98, N 4.93%. ^1^H NMR (CDCl_3_): δ 8.27 (1*H*, t, J = 6.1 Hz; orthopalladated ring), 6.30–7.60 (m, Ph), 3.54 (3*H*, s, CH_3_; major isomer), 3.45 (3*H*, s, CH_3_; minor isomer), 2.29 (3*H*, s, CH_3_; major isomer), 2.07 (3*H*, s, CH_3_; minor isomer). ^31^P NMR (CDCl_3_): δ 132.17 (major isomer), 124.70 (minor isomer).

### Refinement

All the carbon-bonded H atoms were positioned geometrically and refined using a riding model with aromatic C—H = 0.95Å and *U*_iso_(H) = 1.2*U*_eq_(C). The methyl groups were refined with C—H = 0.98Å and *U*_iso_(H) = 1.5*U*_eq_(C). One of the phenyl rings is disordered over two sites with a site occupation factor of 0.512 (8) for the major occupied site. The highest residual peak and the deepest hole in the final difference map are located 0.73 and 0.80Å from the C32 and H26B atom, respectively.

### Crystal data

**Table:**

[Pd(C_18_H_14_O_3_P)Cl(C_5_H_8_N_2_)]	*Z* = 2
*M**_r_* = 547.25	*F*(000) = 552
Triclinic, *P*1	*D*_x_ = 1.586 Mg m^−^^3^
Hall symbol: -P 1	Mo *K*α radiation, λ = 0.71073 Å
*a* = 9.212 (4) Å	Cell parameters from 8960 reflections
*b* = 9.370 (4) Å	θ = 5.0–27.5°
*c* = 14.295 (5) Å	µ = 1.02 mm^−^^1^
α = 85.30 (3)°	*T* = 100 K
β = 84.83 (3)°	Plate, colorless
γ = 69.08 (3)°	0.34 × 0.16 × 0.12 mm
*V* = 1146.2 (8) Å^3^	

### Data collection

**Table:**

Kuma KM-4 CCD diffractometer	5113 independent reflections
Radiation source: fine-focus sealed tube	4855 reflections with *I* > 2σ(*I*)
graphite	*R*_int_ = 0.023
ω scans	θ_max_ = 27.5°, θ_min_ = 5.0°
Absorption correction: analytical (*CrysAlis RED*; Oxford Diffraction, 2010)	*h* = −11→11
*T*_min_ = 0.812, *T*_max_ = 0.908	*k* = −12→10
12801 measured reflections	*l* = −18→18

### Refinement

**Table:**

Refinement on *F*^2^	Primary atom site location: heavy-atom method
Least-squares matrix: full	Secondary atom site location: difference Fourier map
*R*[*F*^2^ > 2σ(*F*^2^)] = 0.029	Hydrogen site location: inferred from neighbouring sites
*wR*(*F*^2^) = 0.082	H-atom parameters constrained
*S* = 1.37	*w* = 1/[σ^2^(*F*_o_^2^) + (0.0102*P*)^2^ + 2.9228*P*] where *P* = (*F*_o_^2^ + 2*F*_c_^2^)/3
5113 reflections	(Δ/σ)_max_ = 0.001
319 parameters	Δρ_max_ = 0.39 e Å^−^^3^
0 restraints	Δρ_min_ = −0.40 e Å^−^^3^

### Special details

**Table:**

Experimental. The crystal was placed in the cold stream of an open-flow nitrogen cryostat (Cosier & Glazer, 1986) operating at 100 K. Analytical numeric absorption correction was carried out with *CrysAlis RED* (Oxford Diffraction, 2010) using a multifaceted crystal model (Clark & Reid, 1995).
Geometry. All e.s.d.'s (except the e.s.d. in the dihedral angle between two l.s. planes) are estimated using the full covariance matrix. The cell e.s.d.'s are taken into account individually in the estimation of e.s.d.'s in distances, angles and torsion angles; correlations between e.s.d.'s in cell parameters are only used when they are defined by crystal symmetry. An approximate (isotropic) treatment of cell e.s.d.'s is used for estimating e.s.d.'s involving l.s. planes.
Refinement. Refinement of *F*^2^ against ALL reflections. The weighted *R*-factor *wR* and goodness of fit *S* are based on *F*^2^, conventional *R*-factors *R* are based on *F*, with *F* set to zero for negative *F*^2^. The threshold expression of *F*^2^ > 2σ(*F*^2^) is used only for calculating *R*-factors(gt) *etc*. and is not relevant to the choice of reflections for refinement. *R*-factors based on *F*^2^ are statistically about twice as large as those based on *F* and *R*- factors based on ALL data will be even larger.

### Fractional atomic coordinates and isotropic or equivalent isotropic displacement parameters (Å^2^)

**Table:**

	*x*	*y*	*z*	*U*_iso_*/*U*_eq_	Occ. (<1)
Pd	0.17867 (3)	0.19205 (3)	0.287224 (16)	0.01206 (7)	
Cl	0.37905 (9)	0.25690 (9)	0.34805 (6)	0.01847 (16)	
P	0.32350 (9)	0.07995 (9)	0.16750 (6)	0.01384 (16)	
O1	0.2280 (3)	−0.0022 (3)	0.11667 (16)	0.0180 (5)	
O2	0.3945 (3)	0.1646 (3)	0.08571 (17)	0.0218 (5)	
O3	0.4854 (2)	−0.0534 (2)	0.18349 (16)	0.0173 (5)	
C11	0.0240 (4)	0.1235 (3)	0.2318 (2)	0.0150 (6)	
C12	0.0744 (4)	0.0299 (3)	0.1564 (2)	0.0150 (6)	
C13	−0.0176 (4)	−0.0347 (4)	0.1165 (2)	0.0209 (7)	
H13	0.0237	−0.1021	0.0668	0.025*	
C14	−0.1716 (4)	0.0014 (4)	0.1510 (3)	0.0226 (7)	
H14	−0.2374	−0.0405	0.1244	0.027*	
C15	−0.2290 (4)	0.0983 (4)	0.2240 (3)	0.0212 (7)	
H15	−0.3349	0.1245	0.2467	0.025*	
C16	−0.1326 (4)	0.1576 (4)	0.2644 (2)	0.0172 (6)	
H16	−0.1735	0.2227	0.3153	0.021*	
C21	0.3189 (4)	0.3137 (4)	0.0487 (2)	0.0192 (7)	
C22A	0.2635 (12)	0.3470 (9)	−0.0393 (6)	0.0265 (18)	0.512 (8)
H22A	0.2646	0.2664	−0.0760	0.032*	0.512 (8)
C23A	0.2057 (12)	0.4969 (9)	−0.0752 (6)	0.0266 (18)	0.512 (8)
H23A	0.1703	0.5151	−0.1369	0.032*	0.512 (8)
C22B	0.2028 (10)	0.3323 (9)	−0.0075 (6)	0.0205 (16)	0.488 (8)
H22B	0.1667	0.2512	−0.0155	0.025*	0.488 (8)
C23B	0.1383 (11)	0.4748 (10)	−0.0532 (6)	0.0242 (18)	0.488 (8)
H23B	0.0588	0.4931	−0.0954	0.029*	0.488 (8)
C24	0.1979 (5)	0.6019 (5)	−0.0343 (4)	0.0454 (13)	
H24	0.1632	0.7006	−0.0654	0.054*	
C25A	0.2420 (8)	0.5870 (8)	0.0695 (5)	0.0246 (18)	0.512 (8)
H25A	0.2305	0.6729	0.1044	0.030*	0.512 (8)
C26A	0.2995 (8)	0.4382 (8)	0.1067 (6)	0.0235 (18)	0.512 (8)
H26A	0.3260	0.4183	0.1704	0.028*	0.512 (8)
C25B	0.3224 (9)	0.5607 (8)	0.0083 (5)	0.0251 (19)	0.488 (8)
H25B	0.3752	0.6314	0.0077	0.030*	0.488 (8)
C26B	0.3876 (8)	0.4183 (8)	0.0560 (5)	0.0179 (17)	0.488 (8)
H26B	0.4758	0.3959	0.0919	0.022*	0.488 (8)
C31	0.4900 (4)	−0.1763 (4)	0.2486 (2)	0.0176 (6)	
C32	0.4921 (5)	−0.3100 (4)	0.2149 (3)	0.0357 (10)	
H32	0.4843	−0.3176	0.1498	0.043*	
C33	0.5056 (7)	−0.4331 (5)	0.2778 (3)	0.0476 (13)	
H33	0.5091	−0.5271	0.2555	0.057*	
C34	0.5142 (5)	−0.4215 (4)	0.3726 (3)	0.0315 (9)	
H34	0.5214	−0.5065	0.4154	0.038*	
C35	0.5123 (4)	−0.2865 (4)	0.4047 (3)	0.0250 (7)	
H35	0.5190	−0.2786	0.4699	0.030*	
C36	0.5005 (4)	−0.1614 (4)	0.3424 (2)	0.0218 (7)	
H36	0.4997	−0.0680	0.3642	0.026*	
N41	0.0314 (3)	0.2786 (3)	0.40502 (19)	0.0162 (5)	
C42	0.0513 (4)	0.2164 (4)	0.4918 (2)	0.0173 (6)	
N43	−0.0556 (3)	0.3072 (3)	0.5523 (2)	0.0215 (6)	
C44	−0.1489 (4)	0.4326 (4)	0.5023 (3)	0.0247 (7)	
H44	−0.2346	0.5153	0.5266	0.030*	
C45	−0.0937 (4)	0.4145 (4)	0.4108 (2)	0.0214 (7)	
H45	−0.1345	0.4839	0.3593	0.026*	
C46	0.1716 (4)	0.0676 (4)	0.5190 (3)	0.0248 (7)	
H46A	0.2526	0.0860	0.5510	0.037*	
H46B	0.2184	0.0114	0.4625	0.037*	
H46C	0.1232	0.0074	0.5614	0.037*	
C47	−0.0743 (5)	0.2778 (5)	0.6540 (3)	0.0351 (9)	
H47A	−0.1217	0.1991	0.6667	0.053*	
H47B	−0.1417	0.3723	0.6829	0.053*	
H47C	0.0278	0.2423	0.6806	0.053*	

### Atomic displacement parameters (Å^2^)

**Table:**

	*U*^11^	*U*^22^	*U*^33^	*U*^12^	*U*^13^	*U*^23^
Pd	0.01199 (11)	0.01131 (11)	0.01229 (12)	−0.00371 (8)	0.00158 (8)	−0.00174 (8)
Cl	0.0169 (3)	0.0196 (4)	0.0210 (4)	−0.0083 (3)	0.0018 (3)	−0.0075 (3)
P	0.0139 (4)	0.0121 (4)	0.0140 (4)	−0.0037 (3)	0.0029 (3)	−0.0007 (3)
O1	0.0159 (11)	0.0207 (11)	0.0161 (11)	−0.0048 (9)	0.0028 (9)	−0.0058 (9)
O2	0.0257 (12)	0.0151 (11)	0.0198 (12)	−0.0046 (9)	0.0103 (10)	0.0012 (9)
O3	0.0136 (10)	0.0136 (10)	0.0230 (12)	−0.0038 (8)	0.0031 (9)	0.0001 (9)
C11	0.0141 (14)	0.0149 (14)	0.0156 (14)	−0.0049 (11)	−0.0022 (11)	0.0028 (12)
C12	0.0144 (14)	0.0152 (14)	0.0137 (14)	−0.0040 (11)	−0.0001 (11)	0.0022 (12)
C13	0.0262 (17)	0.0216 (16)	0.0154 (15)	−0.0087 (14)	−0.0018 (13)	−0.0024 (13)
C14	0.0221 (17)	0.0251 (17)	0.0249 (17)	−0.0131 (14)	−0.0060 (14)	0.0014 (14)
C15	0.0161 (15)	0.0214 (16)	0.0269 (18)	−0.0082 (13)	−0.0014 (13)	0.0020 (14)
C16	0.0166 (15)	0.0151 (14)	0.0184 (15)	−0.0044 (12)	0.0019 (12)	−0.0015 (12)
C21	0.0191 (15)	0.0187 (16)	0.0175 (15)	−0.0059 (12)	0.0039 (12)	0.0026 (13)
C22A	0.041 (5)	0.020 (4)	0.019 (4)	−0.012 (3)	0.008 (4)	−0.005 (3)
C23A	0.033 (5)	0.022 (4)	0.017 (4)	−0.002 (3)	0.000 (3)	0.006 (3)
C22B	0.020 (4)	0.020 (4)	0.023 (4)	−0.009 (3)	0.000 (3)	−0.001 (3)
C23B	0.020 (4)	0.030 (4)	0.022 (4)	−0.008 (3)	−0.003 (3)	0.005 (3)
C24	0.027 (2)	0.037 (3)	0.062 (3)	−0.0071 (18)	0.000 (2)	0.034 (2)
C25A	0.025 (4)	0.018 (3)	0.032 (4)	−0.009 (3)	−0.003 (3)	−0.003 (3)
C26A	0.020 (4)	0.019 (3)	0.030 (4)	−0.006 (3)	−0.007 (3)	0.000 (3)
C25B	0.035 (4)	0.022 (4)	0.022 (4)	−0.016 (3)	−0.001 (3)	0.003 (3)
C26B	0.023 (4)	0.021 (3)	0.011 (3)	−0.010 (3)	−0.003 (3)	0.001 (3)
C31	0.0125 (14)	0.0144 (14)	0.0250 (17)	−0.0040 (11)	−0.0034 (12)	0.0035 (13)
C32	0.060 (3)	0.0213 (18)	0.032 (2)	−0.0178 (18)	−0.025 (2)	0.0042 (16)
C33	0.084 (4)	0.022 (2)	0.049 (3)	−0.028 (2)	−0.038 (3)	0.0105 (19)
C34	0.037 (2)	0.0216 (18)	0.039 (2)	−0.0123 (16)	−0.0185 (18)	0.0119 (16)
C35	0.0239 (17)	0.0234 (17)	0.0231 (17)	−0.0028 (14)	−0.0045 (14)	0.0020 (14)
C36	0.0235 (17)	0.0168 (15)	0.0232 (17)	−0.0047 (13)	0.0000 (13)	−0.0034 (13)
N41	0.0158 (12)	0.0161 (13)	0.0165 (13)	−0.0053 (10)	0.0010 (10)	−0.0036 (10)
C42	0.0157 (15)	0.0180 (15)	0.0183 (15)	−0.0060 (12)	0.0014 (12)	−0.0030 (12)
N43	0.0237 (14)	0.0236 (14)	0.0147 (13)	−0.0057 (12)	0.0033 (11)	−0.0035 (11)
C44	0.0250 (17)	0.0212 (17)	0.0212 (17)	−0.0006 (14)	0.0031 (14)	−0.0028 (14)
C45	0.0211 (16)	0.0174 (16)	0.0217 (17)	−0.0019 (13)	0.0000 (13)	−0.0028 (13)
C46	0.0254 (18)	0.0217 (17)	0.0205 (17)	−0.0012 (14)	0.0006 (14)	0.0026 (14)
C47	0.042 (2)	0.038 (2)	0.0152 (17)	−0.0047 (18)	0.0085 (16)	−0.0018 (16)

### Geometric parameters (Å, °)

**Table:**

Pd—C11	2.003 (3)	C24—H24	0.9500
Pd—N41	2.088 (3)	C25A—C26A	1.381 (11)
Pd—P	2.1667 (12)	C25A—H25A	0.9500
Pd—Cl	2.3890 (12)	C26A—H26A	0.9500
P—O2	1.582 (2)	C25B—C26B	1.400 (11)
P—O3	1.588 (2)	C25B—H25B	0.9500
P—O1	1.607 (3)	C26B—H26B	0.9500
O1—C12	1.412 (4)	C31—C32	1.373 (5)
O2—C21	1.403 (4)	C31—C36	1.375 (5)
O3—C31	1.411 (4)	C32—C33	1.379 (5)
C11—C12	1.386 (5)	C32—H32	0.9500
C11—C16	1.404 (5)	C33—C34	1.379 (5)
C12—C13	1.385 (5)	C33—H33	0.9500
C13—C14	1.388 (5)	C34—C35	1.375 (5)
C13—H13	0.9500	C34—H34	0.9500
C14—C15	1.380 (5)	C35—C36	1.390 (5)
C14—H14	0.9500	C35—H35	0.9500
C15—C16	1.388 (5)	C36—H36	0.9500
C15—H15	0.9500	N41—C42	1.326 (4)
C16—H16	0.9500	N41—C45	1.381 (4)
C21—C22B	1.348 (8)	C42—N43	1.345 (4)
C21—C26B	1.358 (8)	C42—C46	1.486 (4)
C21—C22A	1.370 (8)	N43—C44	1.372 (4)
C21—C26A	1.437 (8)	N43—C47	1.464 (4)
C22A—C23A	1.385 (11)	C44—C45	1.361 (4)
C22A—H22A	0.9500	C44—H44	0.9500
C23A—C24	1.162 (10)	C45—H45	0.9500
C23A—H23A	0.9500	C46—H46A	0.9800
C22B—C23B	1.386 (11)	C46—H46B	0.9800
C22B—H22B	0.9500	C46—H46C	0.9800
C23B—C24	1.530 (10)	C47—H47A	0.9800
C23B—H23B	0.9500	C47—H47B	0.9800
C24—C25A	1.556 (10)	C47—H47C	0.9800
C24—C25B	1.267 (10)		
			
P—Pd—Cl	94.52 (4)	C26A—C25A—C24	114.3 (7)
P—Pd—C11	81.51 (10)	C26A—C25A—H25A	122.9
C11—Pd—N41	93.91 (12)	C24—C25A—H25A	122.9
N41—Pd—Cl	89.88 (8)	C25A—C26A—C21	119.7 (7)
C11—Pd—Cl	175.48 (8)	C25A—C26A—H26A	120.1
N41—Pd—P	173.55 (8)	C21—C26A—H26A	120.1
O2—P—O3	93.7 (2)	C26B—C25B—C24	124.5 (7)
O2—P—O1	105.7 (2)	C24—C25B—H25B	117.8
O3—P—O1	103.4 (2)	C26B—C25B—H25B	117.8
O3—P—Pd	119.78 (9)	C21—C26B—C25B	116.9 (7)
O2—P—Pd	124.07 (9)	C21—C26B—H26B	121.5
O1—P—Pd	107.73 (9)	C25B—C26B—H26B	121.5
C12—O1—P	113.1 (2)	C32—C31—C36	122.2 (4)
C21—O2—P	125.1 (2)	C32—C31—O3	118.3 (3)
C31—O3—P	119.2 (2)	C36—C31—O3	119.4 (3)
C12—C11—C16	115.9 (3)	C31—C32—C33	118.4 (4)
C12—C11—Pd	118.3 (2)	C31—C32—H32	120.8
C16—C11—Pd	125.8 (2)	C33—C32—H32	120.8
C13—C12—C11	123.9 (3)	C32—C33—C34	120.9 (4)
C13—C12—O1	117.2 (3)	C32—C33—H33	119.5
C11—C12—O1	118.9 (3)	C34—C33—H33	119.5
C12—C13—C14	118.4 (3)	C35—C34—C33	119.7 (4)
C12—C13—H13	120.8	C35—C34—H34	120.2
C14—C13—H13	120.8	C33—C34—H34	120.2
C15—C14—C13	120.0 (3)	C34—C35—C36	120.4 (4)
C15—C14—H14	120.0	C34—C35—H35	119.8
C13—C14—H14	120.0	C36—C35—H35	119.8
C14—C15—C16	120.3 (3)	C31—C36—C35	118.4 (4)
C14—C15—H15	119.9	C31—C36—H36	120.8
C16—C15—H15	119.9	C35—C36—H36	120.8
C15—C16—C11	121.5 (3)	C42—N41—C45	106.8 (3)
C15—C16—H16	119.2	C42—N41—Pd	124.8 (3)
C11—C16—H16	119.2	C45—N41—Pd	128.1 (3)
C22B—C21—C26B	124.7 (5)	N41—C42—N43	109.9 (3)
C22B—C21—O2	116.3 (4)	N41—C42—C46	125.5 (3)
C26B—C21—O2	117.5 (4)	N43—C42—C46	124.6 (3)
C22A—C21—O2	123.8 (4)	C42—N43—C44	108.4 (3)
C26A—C21—O2	117.7 (4)	C42—N43—C47	126.7 (3)
C22A—C21—C26A	118.5 (5)	C44—N43—C47	124.9 (3)
C21—C22A—C23A	120.6 (7)	C45—C44—N43	106.1 (3)
C21—C22A—H22A	119.7	C45—C44—H44	127.0
C23A—C22A—H22A	119.7	N43—C44—H44	127.0
C24—C23A—C22A	123.9 (7)	C44—C45—N41	108.8 (3)
C24—C23A—H23A	118.0	C44—C45—H45	125.6
C22A—C23A—H23A	118.0	N41—C45—H45	125.6
C21—C22B—C23B	117.2 (7)	C42—C46—H46A	109.5
C21—C22B—H22B	121.4	C42—C46—H46B	109.5
C23B—C22B—H22B	121.4	H46A—C46—H46B	109.5
C22B—C23B—C24	118.6 (7)	C42—C46—H46C	109.5
C22B—C23B—H23B	120.7	H46A—C46—H46C	109.5
C24—C23B—H23B	120.7	H46B—C46—H46C	109.5
C23B—C24—C25B	116.2 (5)	N43—C47—H47A	109.5
C23A—C24—C25A	122.5 (5)	N43—C47—H47B	109.5
C23A—C24—H24	118.7	H47A—C47—H47B	109.5
C25B—C24—H24	118.5	N43—C47—H47C	109.5
C23B—C24—H24	123.3	H47A—C47—H47C	109.5
C25A—C24—H24	118.7	H47B—C47—H47C	109.5
			
C11—Pd—P—O2	118.0 (2)	C26B—C21—C22B—C23B	−7.2 (12)
Cl—Pd—P—O2	−64.2 (2)	O2—C21—C22B—C23B	−173.0 (6)
C11—Pd—P—O3	−123.6 (2)	C21—C22B—C23B—C24	−2.3 (12)
Cl—Pd—P—O3	54.2 (1)	C22A—C23A—C24—C25A	−4.1 (12)
C11—Pd—P—O1	−6.0 (2)	C22B—C23B—C24—C25B	12.9 (12)
Cl—Pd—P—O1	171.79 (9)	C23A—C24—C25A—C26A	3.6 (9)
O2—P—O1—C12	−129.3 (2)	C24—C25A—C26A—C21	2.2 (9)
O3—P—O1—C12	132.9 (2)	C22A—C21—C26A—C25A	−7.2 (9)
Pd—P—O1—C12	5.2 (2)	O2—C21—C26A—C25A	172.6 (6)
O3—P—O2—C21	−167.0 (3)	C22B—C21—C26B—C25B	6.3 (9)
O1—P—O2—C21	88.0 (3)	O2—C21—C26B—C25B	171.9 (6)
Pd—P—O2—C21	−36.9 (3)	C24—C25B—C26B—C21	6.0 (12)
O2—P—O3—C31	−175.5 (2)	P—O3—C31—C32	99.1 (3)
O1—P—O3—C31	−68.4 (3)	P—O3—C31—C36	−84.2 (3)
Pd—P—O3—C31	51.4 (3)	C36—C31—C32—C33	−0.2 (6)
N41—Pd—C11—C12	−168.8 (2)	O3—C31—C32—C33	176.3 (4)
P—Pd—C11—C12	6.7 (2)	C31—C32—C33—C34	1.1 (8)
N41—Pd—C11—C16	9.0 (3)	C32—C33—C34—C35	−1.3 (7)
P—Pd—C11—C16	−175.6 (3)	C33—C34—C35—C36	0.5 (6)
C16—C11—C12—C13	−3.1 (5)	C32—C31—C36—C35	−0.5 (5)
Pd—C11—C12—C13	174.9 (2)	O3—C31—C36—C35	−177.0 (3)
C16—C11—C12—O1	176.5 (3)	C34—C35—C36—C31	0.4 (5)
Pd—C11—C12—O1	−5.5 (4)	C11—Pd—N41—C42	109.1 (3)
P—O1—C12—C13	179.3 (3)	Cl—Pd—N41—C42	−68.4 (3)
P—O1—C12—C11	−0.3 (3)	C11—Pd—N41—C45	−77.3 (3)
C11—C12—C13—C14	3.0 (5)	Cl—Pd—N41—C45	105.2 (3)
O1—C12—C13—C14	−176.7 (3)	C45—N41—C42—N43	−0.2 (4)
C12—C13—C14—C15	−0.7 (5)	Pd—N41—C42—N43	174.6 (2)
C13—C14—C15—C16	−1.2 (5)	C45—N41—C42—C46	179.0 (3)
C14—C15—C16—C11	1.0 (5)	Pd—N41—C42—C46	−6.3 (5)
C12—C11—C16—C15	1.0 (4)	N41—C42—N43—C44	0.4 (4)
Pd—C11—C16—C15	−176.8 (2)	C46—C42—N43—C44	−178.7 (3)
P—O2—C21—C22B	−74.0 (6)	N41—C42—N43—C47	178.8 (4)
P—O2—C21—C26B	119.1 (6)	C46—C42—N43—C47	−0.3 (6)
P—O2—C21—C22A	−109.2 (6)	C42—N43—C44—C45	−0.5 (4)
P—O2—C21—C26A	70.9 (6)	C47—N43—C44—C45	−179.0 (4)
O2—C21—C22A—C23A	−172.8 (6)	N43—C44—C45—N41	0.5 (4)
C26A—C21—C22A—C23A	7.0 (12)	C42—N41—C45—C44	−0.2 (4)
C21—C22A—C23A—C24	−1.3 (12)	Pd—N41—C45—C44	−174.7 (2)

### Hydrogen-bond geometry (Å, °)

**Table:**

*D*—H···*A*	*D*—H	H···*A*	*D*···*A*	*D*—H···*A*
C14—H14···O3^i^	0.95	2.66	3.365 (4)	132
C15—H15···Cl^i^	0.95	2.80	3.720 (4)	162
C33—H33···Cl^ii^	0.95	2.88	3.541 (4)	128
C35—H35···Cl^iii^	0.95	2.89	3.817 (4)	164
C44—H44···Cl^iv^	0.95	2.78	3.651 (4)	154

Symmetry codes: (i) *x*−1, *y*, *z*; (ii) *x*, *y*−1, *z*; (iii) −*x*+1, −*y*, −*z*+1; (iv) −*x*, −*y*+1, −*z*+1.
